# Expanding the Malaria Antibody Toolkit: Development and Characterisation of *Plasmodium falciparum* RH5, CyRPA, and CSP Recombinant Human Monoclonal Antibodies

**DOI:** 10.3389/fcimb.2022.901253

**Published:** 2022-06-16

**Authors:** Adéla Nacer, Gaily Kivi, Raini Pert, Erkki Juronen, Pavlo Holenya, Eduardo Aliprandini, Rogerio Amino, Olivier Silvie, Doris Quinkert, Yann Le Duff, Matthew Hurley, Ulf Reimer, Andres Tover, Simon J. Draper, Sarah Gilbert, Mei Mei Ho, Paul W. Bowyer

**Affiliations:** ^1^Division of Bacteriology, National Institute for Biological Standards and Control (NIBSC), Medicines and Healthcare products Regulatory Agency (MHRA), Potters Bar, United Kingdom; ^2^Icosagen Cell Factory OÜ, Tartumaa, Estonia; ^3^Research and Development, JPT Peptide Technologies GmbH, Berlin, Germany; ^4^Malaria Infection & Immunity Unit, Institut Pasteur, Paris, France; ^5^Sorbonne Université, INSERM, CNRS, Centre d’Immunologie et des Maladies Infectieuses, CIMI-Paris, Paris, France; ^6^Department of Biochemistry, University of Oxford, Oxford, United Kingdom; ^7^Centre for Aids Reagents, National Institute for Biological Standards and Control (NIBSC), Medicines and Healthcare products Regulatory Agency (MHRA), Potters Bar, United Kingdom

**Keywords:** *Plasmodium falciparum*, monoclonal antibodies, standardisation, assay development, malaria, PfRH5, PfCyRPA, PfCSP

## Abstract

Malaria, an infection caused by apicomplexan parasites of the genus *Plasmodium*, continues to exact a significant toll on public health with over 200 million cases world-wide, and annual deaths in excess of 600,000. Considerable progress has been made to reduce malaria burden in endemic countries in the last two decades. However, parasite and mosquito resistance to frontline chemotherapies and insecticides, respectively, highlights the continuing need for the development of safe and effective vaccines. Here we describe the development of recombinant human antibodies to three target proteins from *Plasmodium falciparum*: reticulocyte binding protein homologue 5 (PfRH5), cysteine-rich protective antigen *(Pf*CyRPA), and circumsporozoite protein (*Pf*CSP). All three proteins are key targets in the development of vaccines for blood-stage or pre-erythrocytic stage infections. We have developed potent anti-*Pf*RH5, *Pf*CyRPA and *Pf*CSP monoclonal antibodies that will prove useful tools for the standardisation of assays in preclinical research and the assessment of these antigens in clinical trials. We have generated some very potent anti-*Pf*RH5 and anti-*Pf*CyRPA antibodies with some clones >200 times more potent than the polyclonal anti-AMA-1 antibodies used for the evaluation of blood stage antigens. While the monoclonal and polyclonal antibodies are not directly comparable, the data provide evidence that these new antibodies are very good at blocking invasion. These antibodies will therefore provide a valuable resource and have potential as biological standards to help harmonise pre-clinical malaria research.

## 1 Introduction

Malaria is a vector-borne disease endemic to large parts of the world. It is estimated that nearly half of the global population is at risk with annual deaths of over 627,000 people, primarily African children under 5 years of age (WHO, 2021). The first malaria vaccine for children in areas of moderate to high transmission was recommended by the World Health Organisation (WHO) in October 2021. This recommendation is based on the results from an extended pilot programme of Mosquirix^®^ (RTS,S/AS01) in Ghana, Malawi, and Kenya as part of routine childhood immunisations (Adepoju, 2019) and represents significant progress in the fight against malaria. In addition, a second generation subunit vaccine (R21/Matrix-M; Datoo et al., 2021) received authorisation for a phase 3 clinical trial (NCT04704830). Additionally, phase I clinical trials in controlled human malaria infections with whole attenuated sporozoite vaccines have recently been published (NCT02511054, NCT03083847) with promising results (Mwakingwe-Omari et al., 2021). Finally, a blood-stage vaccine has undergone phase I/IIa clinical trials (Minassian et al., 2021), further adding to the vaccine portfolio.

Malaria is caused by parasites of the genus *Plasmodium* that have a complex life cycle with development in a mosquito vector and in a vertebrate host. In humans, the first step of infection occurs when parasites enter the human host *via* an infected mosquito bite and begin to replicate in the liver. Parasites released from the liver enter the circulation where they replicate within erythrocytes; the intra-erythrocytic cycle is associated with malaria pathology and is the primary target of anti-malarial chemotherapies (Maier et al., 2019). While anti-malarial drugs and control strategies are vital to malaria control efforts, parasite drug resistance and mosquito insecticide resistance are on the rise and pose risks to the gains obtained over the past two decades (WHO, 2021). Furthermore, disruptions in healthcare access caused by the COVID-19 pandemic have increased deaths resulting from malaria infection in 2020 with an estimated 241 million cases and 627,000 deaths (WHO, 2021).

Bearing in mind that eradicating malaria parasites following infection requires protracted treatment regimens with serious socioeconomic consequences, the need for malaria vaccines remains as urgent as ever. Malaria vaccines targeting parasite antigens across all key stages of the parasite life cycle are being developed. These include vaccines blocking transmission to the vector (Challenger et al., 2021) and those targeting liver infection (Mwakingwe-Omari et al., 2021), and blood stage parasites (Vijayan and Chitnis, 2019). In addition, vaccines to prevent placental malaria are also in phase Ia clinical trials (Sirima et al., 2020). The vaccines closest to regulatory approval and licensure, including Mosquirix^®^ (RTS,S/AS01) and R21/Matrix-M, target parasite infection of the liver with other vaccine candidates targeting development of the parasites in red blood cells (Sato, 2021a; Sato, 2021b).

Many assays are available for malaria research, but they are not standardised, making inter-laboratory comparison of data very challenging. Furthermore, the high genetic variability of *P. falciparum* presents an additional, and considerable, challenge for harmonisation of the field, especially in diagnosis and treatment of the disease. Additional challenges exist in the characterisation of immune responses, disease surveillance, and in the spectrum of assays used to understand the disease in terms of management and treatment. The first serological WHO Reference Reagent for *P. falciparum* (Bryan et al., 2017) and the first WHO International Standard for *P. falciparum* antigens (Harris et al., 2017) were major breakthroughs for standardising vaccine assays and diagnostics, respectively. However, reagents that enable standardisation throughout all aspects of preclinical development of malaria vaccine candidates are still needed for reliable quantification of malaria antigens. The evaluation of vaccine efficacy using *in vitro* and *in vivo* assays is not currently harmonised which in turn effects reproducibility, inhibits progress and squanders investment.

Preclinical evaluation of malaria vaccines prior to controlled human malaria infection studies and clinical trials (Chattopadhyay and Pratt, 2017; Good and Miller, 2018; Artaud et al., 2019; Matuschewski and Borrmann, 2019; Lyke et al., 2020) includes standard laboratory assays for malaria blood stages, such as *in vitro* growth inhibition activity (GIA) assay (Malkin et al., 2005) *in vivo* passive antibody transfer in mice as well as lesser used immunogenicity and efficacy studies in non-human primates (Douglas et al., 2015). Variability in these assays can be caused by the community using a number of different reagents, some being commercial or shared, some being used by one or few laboratories, and many being finite (e.g. the anti-AMA-1 polyclonal antibody BG98 (Faber et al., 2013), meaning again that results are not always or comparable across laboratories and over time. The lack of a defined standard makes meaningful comparisons difficult and impedes the development of novel invasion-blocking candidate antigens.

In addition to these problems for assays directed against blood stage malaria parasites, similar challenges exist for assays involved in targeting *P. falciparum* sporozoites, e.g. invasion, cell traversal, opsonic phagocytosis and cytotoxicity assays (Sinnis et al., 2013; Tavares et al., 2013a; Tavares et al., 2013b; Formaglio et al., 2014; Steel et al., 2017; Aliprandini et al., 2018). The ‘gold standard’ control antibody for these assays is a mouse monoclonal antibody named 2A10 that recognises NANP repeats of the *P. falciparum* circumsporozoite protein (*Pf*CSP) (Zavala et al., 1983; Hollingdale et al., 1984; Anker et al., 1990; Zhang et al., 2017). However, the hybridoma cell line used for production of has 2A10 been widely shared and propagated in different laboratories likely leading to a drift in its biochemical and biophysical properties. Differences may arise due to the potential for genetic instability of the hybridomas - as such it is no longer suitable as a standard (Bradbury and Pluckthun, 2015). This potential drift is difficult to quantify in the absence of a primary standard. As such the generation of the recombinant monoclonal antibodies described here provides an opportunity for the development new standards that target *Pf*CSP.

The European Research Infrastructures for Poverty Related Diseases (EURIPRED) was a collaborative research and infrastructure project that aimed to generate, coordinate and integrate resources to support international research on HIV/AIDS, tuberculosis, malaria and Hepatitis B & C. As part of this project we generated recombinant human monoclonal antibodies that could be used to develop and standardise assays for *P. falciparum* malaria vaccine research and development. These materials, intended to support the research and development community, are available from the Centre for AIDS Reagents repository at NIBSC (www.nibsc.org).

We have generated recombinant human monoclonal antibodies directed against the *P. falciparum* blood stage invasion proteins Reticulocyte binding protein Homologue 5 (*Pf*RH5), Cysteine-Rich Protective Antigen (*Pf*CyRPA) and the dominant sporozoite antigen circumsporozoite protein (*Pf*CSP). *Pf*RH5 is a leading blood stage malaria vaccine target and forms a complex with *Pf*CyRPA and *Pf*RH5-interacting protein (*Pf*RIPR) critical for red-cell invasion by merozoites (Healer et al., 2019; Knuepfer et al., 2019; Ragotte et al., 2020; Ndwiga et al., 2021). Antibodies to both antigens are highly effective at inhibiting growth of *P. falciparum* (Dreyer et al., 2012; Douglas et al., 2014; Volz et al., 2016) and such antibodies may provide suitable reference reagents to standardise GIA assays and other erythrocyte invasion assays – by having a standard with defined neutralising activity, to which the neutralising activity of other materials can be compared. We also developed human monoclonal antibodies to *Pf*CSP, the key antigen of the Mosquirix^®^ (Wilby et al., 2012; Adepoju, 2019; Cotton, 2020) and R21 (Datoo et al., 2021) vaccines designed to prevent *P. falciparum* infection in the liver. *Pf*CSP is expressed on the surface of sporozoites and is the main target of the immune response during the initial phase of infection (Zhang et al., 2017).

Here we describe the generation and characterisation of human monoclonal antibodies against key *P. falciparum* antigens and discuss how their use may support malaria vaccine development. We hope that these monoclonal antibodies will facilitate better cross-laboratory comparisons of novel blood and liver stage malaria vaccine candidates.

## 2 Materials and Methods

### *2.1 Plasmodium falciparum* Parasites

*P*. *falciparum* laboratory isolates FCR3 and NF54 were obtained from The European Malaria Reagent Repository (http://www.malariaresearch.eu/). Parasites were maintained at 4% haematocrit in A+ erythrocytes (National Health Service Blood and Transplant, UK) at 5% CO_2_ and atmospheric O_2_ as previously described (Trager and Jensen, 1976).

### 2.2 Immunogens and Immunizations

Recombinant monoclonal antibodies were produced using HybriFree Technology (Icosagen) as previously described (Kivi et al., 2016). Two 4-8 month old chickens (1.7-2.1 kg) and three rabbits (4-5.5 kg) of >4 months old each were immunised with recombinant *Pf*RH5 (Jin et al., 2018) and *Pf*CyRPA (generated in house, Jenner Institute, University of Oxford) and recombinant *Pf*CSP (kindly provided by D. Narum, NIAID, NIH, DHHS) as previously described (Kivi et al., 2016). Rabbits and chickens were immunised in parallel as antigens elicit different responses in different species thereby increasing the likelihood of identifying antibodies of interest. Briefly, 0.2 mg of antigen per test animal per injection was used. Rabbits received four subscapular immunisations at approximately 3 week intervals. Chickens were immunised intramuscularly three times with at ~2 week intervals. The primary immunisation was performed in Freund's complete adjuvant, while all subsequent immunisations were with Freund's incomplete adjuvant (IFA). Booster doses were performed as two separate injections, where the first (0.1 mg) was administered by subscapular injection (rabbits) or intramuscularly (chickens) in Freund's incomplete adjuvant and the second (0.1 mg) was administered intravenously in phosphate buffered saline (PBS) – both injections were given in this way to all test animals and at the same time point (Kivi et al., 2016). Spleens were isolated 3 days after the final boost, homogenized in ice-cold PBS, and cryopreserved in heat inactivated foetal bovine serum with 10% DMSO prior to long-term storage in liquid nitrogen (Kivi et al., 2016). All procedures on animals were performed in compliance with European Union directive 86/609/EEC and approved by the Estonian National Board of Animal Experiments (No. 115, 07.09.2012; No. 87, 28.08.2007).

### 2.3 Production of Human Recombinant Monoclonal Antibodies

Recombinant monoclonal antibodies were generated as previously described (Kivi et al., 2016). Briefly, panning of 2 x 10^4^ splenic cells from immunised animals was performed on antigen-coated (5 μg/ml) immune modules (ThermoFisher Scientific) for 45 min after which unbound cells were removed by washing in PBS. RNA was extracted from the bound cells, reverse transcribed in cDNA using the Superscript IV First-Strand Synthesis System (Invitrogen), which was subsequently used as a template to amplify by PCR the variable light (VL) and heavy (VH) chains.

Amplified VL and VH chains were cloned into human immunoglobulin G1 (hIgG1) expression vectors by circular polymerase extension cloning (CPEC) (Quan and Tian, 2009). *Escherichia coli* DH5α were transformed with hIgG1 expression vector pools and grown in liquid medium on a shaker at 37°C overnight. Plasmid DNA was extracted, purified, and transfected into a transgenic Chinese hamster ovary (CHO) cell line named CHOEBNALT85-1E9 for hIgG1 pool production as previously described (Kivi et al., 2016). Single clones were selected on Luria Broth ampicillin agar medium. 48- 72 hours after transfection, recombinant human IgG1 (rIgG1) pools were tested in enzyme linked immunosorbent assay (ELISA). Maxisorp Immuno modules (Thermo Fisher Scientific) were coated with the specific antigen at 1 µg/ml in PBS overnight. Plates were washed 4 times and blocked with PBS containing 2% bovine serum albumin (BSA) and 0.05% Tween-20. Colonies from ELISA positive pools were grown in liquid medium overnight in a shaker at 37°C and in a 96-well microtiter plate. Plasmid DNA was isolated and transfected into CHOEBNALT85-1E9 cells for transient antibody production and cell culture supernatants were collected after 48 - 72 h and analysed by ELISA to confirm antigen specific binding. Positive clones were then sequenced for VH/VL sequence verification using the international ImmunoGeneTics (IMGT) information system (Lefranc et al., 1999; Giudicelli et al., 2005; Lefranc, 2014; Lefranc et al., 2015).

Plasmid clones were transfected into CHOEBNALT85-1E9 for transient production in 2 ml of media. Ten days after transfection, cells were removed by centrifugation (300*g* for 5 min) and 1.5 ml of each supernatant was aliquoted into microtubes. The concentration of the antibodies produced in crude cell supernatant was measured by biolayer interferometry (Octet^®^ K2 System; ForteBio) using Protein A sensors and appropriate human IgG1 standards. The supernatants were stored at -80°C until further testing. Cell culture supernatants containing anti-*Pf*RH5 and anti-*Pf*CyRPA antibodies were individually screened using the red cell invasion assay to assess functional activity. Selected *Pf*RH5 and *Pf*CyRPA mAbs were then expressed and purified on a small scale to confirm inhibitory activity, followed by scale up to 400 ml cultures of CHOEBNALT85-1E9 cells and purified by HiTrap MabSelect SuRe affinity chromatography with buffer exchange using HiTrap Desalting Columns to PBS pH 7.4 (GE Healthcare). Purified antibodies were adjusted to a concentration of 1 mg/ml, analysed by ELISA and non-reduced gel electrophoresis, filtered, aliquoted and stored at -75°C; no additives or stabilising reagents were added to the purified antibodies to prevent interference in the functional assays. Culture supernatants containing anti-*Pf*CSP mAbs were used for initial screenings and concentrations determined as described above for the *Pf*Rh5 and *Pf*CyRPA antibodies. Further work to express and purify anti-*Pf*CSP mAbs is on-going. All mAbs described here are available from the Centre for AIDS Reagents repository at NIBSC (www.nibsc.org).

### 2.4 Blocking Activity of *Pf*RH5 and *Pf*CyRPA Recombinant IgGs

*Pf*RH5 and *Pf*CyRPA form a complex with *Pf*RIPR during merozoite invasion of erythrocytes (Healer et al., 2019; Knuepfer et al., 2019; Ndwiga et al., 2021). The formation of the complex is required for erythrocyte invasion (Volz et al., 2016). Biolayer interferometry (Octet^®^ K2 System, Pall ForteBio) (Li et al., 2017) was performed to determine the potential of the mAbs to block *Pf*CyRPA-*Pf*RH5 interactions. Monoclonal antibody cell culture supernatants were incubated with immobilised recombinant proteins to measure the dissociation of antigen-antibody and infer antibody blocking activity.

Recombinant *Pf*CyRPA molecules were conjugated to biotin with a ratio of 1:5 using Thermo Scientific™ EZ-Link™ NHS-PEG4-Biotin, No-Weigh™ Format (A39259). Two streptavidin (SA) sensors were incubated in kinetic buffer (PBS containing 0.1% BSA, 0.02% Tween-20, and 0.1% Proclin™ 300) for 11 min in plates with shaking at 1000 rpm to determine the baseline. The two sensors were then used to immobilise biotinylated *Pf*CyRPA by incubation in kinetic buffer containing *Pf*CyRPA-biotin (2 μg/ml) for 11 min at 1000 rpm. The sensors were incubated once more in kinetic buffer alone for 11 min at 1000 rpm to reset to baseline and then incubated in cell culture supernatants diluted 1:2 with kinetic buffer for 22 min at 1000 rpm to saturate the immobilised *Pf*CyRPA-biotin molecules with anti-*Pf*CyRPA antibodies. The sensors were incubated again in kinetic buffer alone for 11 min at 1000 rpm. Finally, one sensor was incubated in kinetic buffer and the other in kinetic buffer containing 12 µg/ml (~0.08 nM) recombinant *Pf*RH5 for 33 min at 1000 rpm.

Blocking activity measurements of anti-*Pf*RH5 antibodies were performed with the following differences. Anti-*Pf*RH5 antibody cell culture supernatants were incubated with recombinant *Pf*RH5 (5 μg/ml) for ≥ 1h at room temperature (RT) to achieve a state of equilibrium. In addition, between sample measurements, used sensors were regenerated using glycine buffer (pH 1.7) and kinetic buffer. Finally, one sensor was incubated in negative cell culture supernatant diluted 1:2 with kinetic buffer containing recombinant *Pf*RH5 (5 µg/ml) and the other sensor was incubated in *Pf*RH5 antibody cell culture supernatant diluted 1:2 in kinetic buffer containing 5 μg/ml recombinant *Pf*RH5 for 33 min at 1000 rpm. The operating temperature for the Octet was at 30°C and all other processes performed at RT unless indicated otherwise.

### 2.5 Generation and Validation of *P*. *falciparum* Peptide Microarrays

We generated overlapping 15-mer peptides for 18 P*. falciparum* and 3 P*. vivax* antigens. We included key vaccine antigens for both species (e.g. *Pf*AMA-1, *Pf*CSP, and *Pf*s25 or *Pv*CSP, *Pv*s25, and *Pv*DBP). To ensure we captured sequence diversity for these highly polymorphic antigens we used sequence information available from MalariaGEN (MalariaGen et al., 2021). We included all mutations found in *P. vivax* and only those with a frequency of >0.5% for *P. falciparum* proteins. The peptides were synthesised and spotted onto microarray slides (RT-HD-Plas, JPT Technologies). Peptides were printed into three individual subarrays per slide and used to screen the polyclonal sera, culture supernatants containing the mAbs, and purified mAbs. Briefly, samples were diluted 1:2000 for cell culture supernatants or used at 50 µg/ml for purified recombinant mAbs in Superblock T20 buffer (Thermo/Pierce, #37516); 300 µl was added to the slides and they were incubated for 2 h at 30°C on each of the triplicate arrays. The primary analyte was washed 5 times for 3 min with 2-4 ml of wash buffer (1X Tris buffered saline (TBS) + 0.1% Tween20). The arrays were then incubated with a Cy5-conjugated anti-human IgG1, anti-rabbit IgG, or anti-chicken IgY secondary antibody, as appropriate, diluted to 1 μg/ml in blocking buffer for 45 min at 30°C. Slides were washed again in wash buffer and dried with a gentle stream of nitrogen and stored in the dark until fluorescence scanning on a GenePix 4330A scanner using a scanning resolution of 10 µm per pixel.

### 2.6 Screening by Invasion Assay of Antibodies From Cell Culture Supernatants

#### *2.6.1 Pf*RH5 and *Pf*CyRPA Recombinant IgGs

Crude cell culture supernatants containing anti-*Pf*RH5 and anti-*Pf*CyRPA antibodies were screened by invasion assay to determine their capacity to inhibit invasion of *P. falciparum* FCR3 parasites *in vitro*. Briefly, *P. falciparum* FCR3 mature stage parasites (schizonts) were enriched by magnet purification, diluted into culture medium containing A+ red blood cells at 4% haematocrit and dispensed into 96-well plates. Three dilutions were tested in duplicate for each antibody; 1:5, 1:25, and 1:125. Antibodies were added to each well for a final volume of 125 µl/well. For each plate a serial dilution of BG98 antibodies (anti-*Pf*AMA-1; NIBSC #NR0014) starting at 6 mg/ml was included to provide a standard curve as well as a positive control for the inhibition of merozoite invasion. Cell culture supernatant from non-transfected cells was used a negative control. Finally, a minimum of 8 wells containing no antibodies was included per plate as an additional control. A volume of 25 µl of culture was removed from each well, fixed with PBS containing 1% PFA and 0.05% GTA. Cells were stained with SYBR Green to determine parasitaemia by flow cytometry. A further 25 µl was removed 48 h later to measure parasitaemia and calculate invasion inhibition after one intra-erythrocytic cycle. For the selection of the recombinant mAb candidates, invasion assays were performed as described above with some modifications. Three concentrations were tested in duplicate for each antibody; 16.4 µg/ml [112.3 nM], 3.28 µg/ml [22.5 nM], and 0.656 µg/ml [4.5 nM]. These concentrations, based on the antibodies with the lowest concentrations within the crude cell supernatant, were selected to enable comparisons at set concentrations across all antibodies.

#### *2.6.2 Pf*CSP Recombinant IgGs

Sporozoite cell traversal assays were performed using Hepa1-6 cells (ATCC CRL-1830) and *P. falciparum* NF54 sporozoites (*Pf*NF54) isolated by hand dissection of infected *A. stephensi* mosquitoes (provided by the Department of Medical Microbiology, University Medical Centre, St Radboud, Nijmegen, the Netherlands). Hepa1-6 cells were seeded at 3 x 10^4^/well in 96 well plates and cultured at 37°C under 5% CO_2_ in DMEM medium (41966029, Gibco) supplemented with 10% (v/v) foetal calf serum (10500064, Gibco), 2 mM L-glutamine (25030024, Thermo Scientific), and 1% (v/v) of a penicillin–streptomycin solution (15140122, Gibco). The following day 5 x 10^4^ *Pf*NF54 sporozoites in complete culture medium were added to the cells in triplicate wells with 1 mg/ml rhodamine-conjugated Dextran (D1817, Molecular Probes) and either 2 or 20 µg/ml of the test antibodies with normal cell culture supernatant as a negative control. Cell cultures were incubated for 3 h at 37°C, then washed, trypsinised, fixed in 1% formaldehyde in PBS, and analysed on a Guava EasyCyte 6/2L bench cytometer equipped with a 532 nm laser (Millipore), for detection and quantification of dextran-positive cells.

Characterisation of antibody binding to sporozoites was performed using 2.5 x 10^4^ P*. falciparum* NF54 sporozoites per sample fixed in 2% PFA in PBS and detected with 2 µg/ml of the rIgG1 diluted in 1% BSA-PBS. Sporozoites were labelled in 15 µl final solution for 1 h on ice before addition of 4 µg/ml AlexaFlour 488 goat anti-human IgG1 (H + L) (Invitrogen, A11013) + 0.5 µg/ml AlexaFluor 647-conjugated 2A10 in 1% BSA-PBS in a final volume of 30 µl. Samples were incubated for 30 min on ice and analysed by flow cytometry on a CytoFLEX S flow cytometer (Beckman Coulter). A minimum of 3000 2A10-positive *Pf*NF54 sporozoites were acquired.

Antibody cytotoxicity was determined as previously described (Aliprandini et al., 2018) using *P. berghei* NK65 sporozoites expressing GFP and a hybrid CSP containing the central repeats region of *P. falciparum* CSP (*PbPf*-GFP). After incubation of 1.2 x 10^4^ sporozoites for 45 min at 37°C with 80 µg/ml of *Pf*CSP antibodies (except for 2G12, which was tested at 60 µg/ml) in HybriMed supernatant and 10% FCS, and with 5 µg/ml of propidium iodide (PI), sporozoites were analysed by flow cytometry. Viable sporozoites were considered as GFP positive and PI negative, and viability was normalized to HybriMed medium without PfCSP antibodies, set to 100% viability.

### 2.7 Characterisation of the Purified Recombinant *Pf*RH5 and *Pf*CyRPA IgGs

Invasion assays were performed as described above. Briefly, *P*. *falciparum* FCR3 or *Pf*NF54 mature stage parasites (schizonts) were enriched by magnet purification, diluted into culture medium containing A+ red blood cells at 4% haematocrit and dispensed into 96-well plates. Three concentrations were tested in duplicate for each antibody; 16.4 µg/ml [112.3 nM], 3.28 µg/ml [22.5 nM], and 0.656 µg/ml [4.5 nM]. Antibodies were added to each well for a final volume of 125 µl/well. For each plate a serial dilution of BG98 antibodies (anti-*Pf*AMA-1; NIBSC #NR0014) starting at 6 mg/ml was included to provide a standard curve as well as a positive control for the inhibition of merozoite invasion. Finally, a minimum of 2 wells without antibodies was included per plate as a negative control. A volume of 25 µl of culture was removed from each well, fixed with PBS containing 1% PFA and 0.05% GTA. Cells were stained with SYBR Green to determine parasitaemia by flow cytometry. A further 25 µl was removed 48 h later to measure parasitaemia and calculate invasion inhibition relative to the no antibody control after one intra-erythrocytic cycle. Statistical analyses were performed using a General Linear Model in Minitab^®^ 21.1.1 after verifying assumptions for a parametric test were met (i.e. equality of variances and normal distribution).

## 3 Results

### 3.1 Generation of *Pf*RH5 and *Pf*CYRPA Recombinant Human IgGs

**Figure 1:**
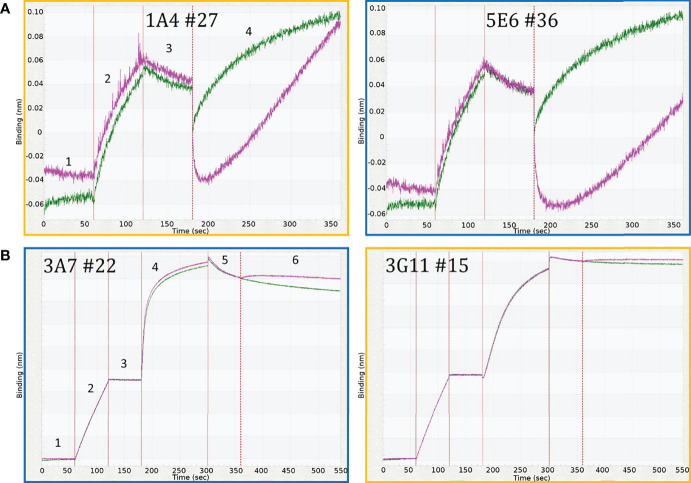
Biolayer interferometry traces of crude hIgG1 clones showing different blocking profiles for **(A)** *Pf*RH5 clones 1A4#27 and 5E6#36; and **(B)** *Pf*CyRPA clones 3A7#22 and 3G11#15. For *Pf*RH5 **(A)** traces show cell culture supernatant containing only *Pf*RH5 recombinant hIgG1 antibodies (green) and cell culture supernatant containing both recombinant *Pf*RH5 (*r*PfRH5) and recombinant human IgG1 antibodies (pink). Antibody 1A4#27 does not block *Pf*RH5 binding to *Pf*CyRPA. Conversely, antibody 5E6#39 blocks binding of *Pf*RH5 to *Pf*CyRPA (step 4). The numbered steps show 1) baseline for both sensors incubated in kinetic buffer; 2) incubation of the sensors with recombinant *Pf*CyRPA-biotin conjugate (*r*PfCyRPA); 3) baseline with the immobilised r*Pf*CyRPA-biotin in kinetic buffer; and 4) association of r*Pf*RH5 to *Pf*CyRPA. For *Pf*CyRPA **(B)** traces show incubation of one sensor in kinetic buffer (green) and incubation of the other sensor (pink) with *r*PfRH5 in association step (step 6). The numbered steps show 1) baseline readings for both sensors in kinetic buffer; 2) incubation with biotin-conjugated recombinant *Pf*CyRPA in kinetic buffer; 3) baseline readings for sensors with immobilised r*Pf*CyRPA in kinetic buffer; 4) saturation of the sensors with crude antibody cell culture supernatant containing specific anti-*Pf*CyRPA recombinant hIgG1s (pink); 5) baseline with immobilized *Pf*CyRPA-antibody complex in kinetic buffer; 6) One sensor incubated in kinetic buffer (green) and the second in kinetic buffer containing PfRH5 (pink). Clone 3G11#15 shows blocking activity (orange outline) while antibody clone 3A7#22 does not block binding of r*Pf*RH5 to r*Pf*CyRPA (blue outline). The y-axis shows binding (nm) and x-axis time (sec).

A total of 64 and 32 panning reactions were performed for *Pf*RH5 from chicken and rabbit splenocytes, respectively (**Table 1**), from which 1034 clones were tested by ELISA and 256 were positive. A total of 194 *Pf*RH5 positive clones were sequenced and of those 33 unique sequences were identified; 26/33 unique sequences were obtained from chicken immunisations and 7/33 from rabbits. Cell culture supernatants from the 33 *Pf*RH5 antibody clones were tested for antigen binding using biolayer interferometry (BLI), which identified 18 *Pf*RH5 mAbs with blocking activity. To do this, biotinylated recombinant r*Pf*RH5 was exposed to its recombinant binding partner r*Pf*CyRPA in the presence or absence of the mAb. **Figure 1** shows examples of two different BLI profiles observed for *P*fRH5 and *Pf*CyRPA recombinant IgG1 clones. The clones shown exhibit either a reduced r*Pf*RH5-r*Pf*CyRPA binding (5E6#36, 3A7#22) or not (1A4#27, 3G11#15. All but one of the *Pf*RH5 mAbs with blocking activity were isolated from chicken.

**Table 1:** Summary of *P. falciparum* RH5 and CyRPA monoclonal antibody generation.

Antigen	Species	Panning Reactions	+ cloning reactions	+ clones (ELISA)	Unique clones (sequencing)	Clones with blocking activity
*Pf*CyRPA	Chicken	64	25	125	12	11
	Rabbit	16	11	14	5	2
*Pf*RH5	Chicken	64	23	240	26	17
	Rabbit	32	9	16	7	1

The table shows the number of independent clones that were generated through key steps in the development of the recombinant monoclonal antibodies. Isolated spleenocytes were panned on the target antigen. + cloning reactions indicates the number of positive clones isolated following cloning into the hIgG1 expression vectors. RNA from bound cells was used for cloning into the expression vector. + clones (ELISA) indicates the number of those clones that bound to the antigen by ELISA. The number of unique clones as identified by sequencing is also shown. The number clones with blocking activities as measured by biolayer interferometry is shown in the last column.

Similarly to *Pf*RH5, 64 panning reactions were performed for *Pf*CyRPA from chicken splenocytes and sixteen from rabbit splenocytes (**Table 1**). Of those, 55 cDNAs were synthesised and antibody expression of positive clones confirmed by ELISA. Of the 1040 clones tested, 139 were positive for *Pf*CyRPA of which 91 were sequenced; 17 unique sequences were identified. Twelve of the unique *Pf*CyRPA sequences were obtained from the chicken immunisations and 5 from rabbits. Again, using the Octet K2 system to evaluate the blocking activity, cell culture supernatants from the 17 *Pf*CyRPA antibody clones were tested. 13 Thirteen had blocking activity (11 from chicken immunisations and 2 from rabbits). Biolayer interferometry traces for *Pf*RH5 and *Pf*CyRPA of rIgG1 monoclonal antibodies with and without blocking activity are shown in **Supplementary Figure 1**.

### 3.2 Selection of *Pf*RH5 and *Pf*CyRPA Recombinant Human IgGs

**Figure 2:**
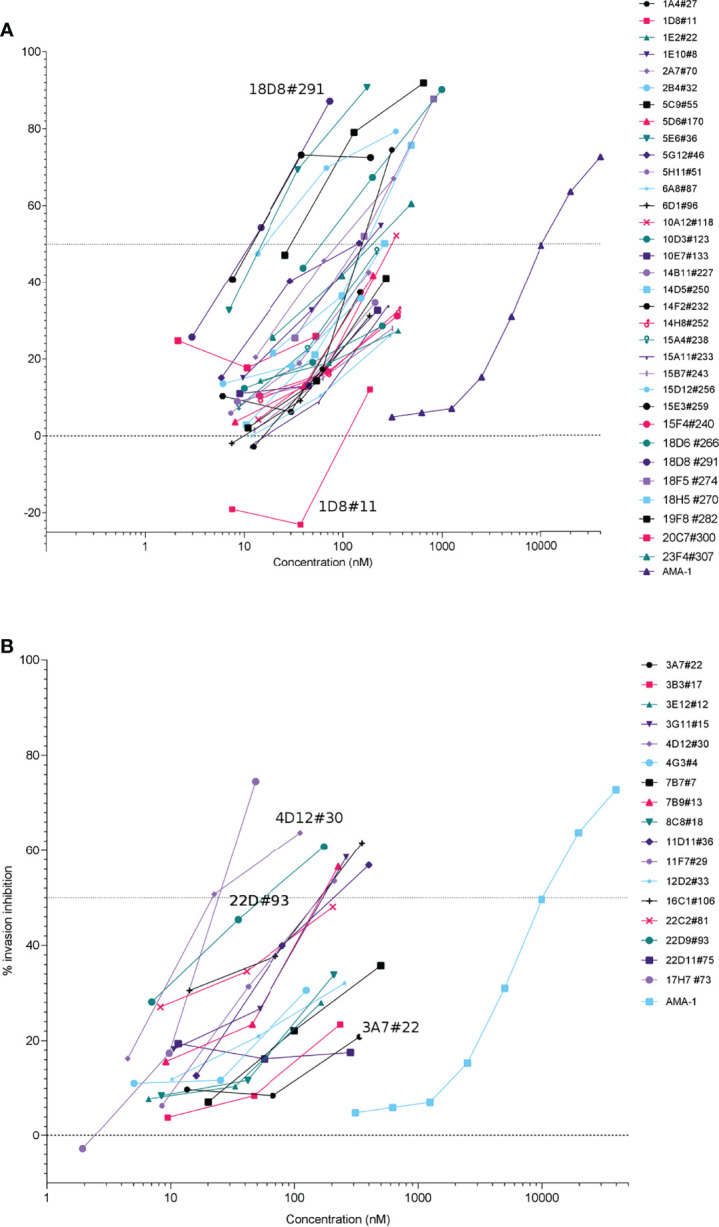
Initial screen of crude cell culture supernatant of expressed anti-*Pf*RH5 **(A)** and anti-*Pf*CyRPA **(B)** antibodies. Antibodies were tested on *P. falciparum* FCR3 during one intra-erythrocytic cycle. Invasion inhibition was determined by measuring parasitaemia by flow cytometry, percentage inhibition was calculated relative to the no antibody control wells. Invasion of parasite by AMA-1 polyclonal antibodies (BG98 standard) is shown for comparison. Many monoclonal antibodies show high potency at low nanomolar (nM) concentrations as compared to polyclonal anti-AMA-1 where the concentration of total IgG is used rather than the antigen-specific fraction.

The ability of the recombinant antibodies to block *P. falciparum* invasion was next tested by screening all 33 *Pf*RH5 clones and 17 *Pf*CyRPA clones with the *in vitro* invasion assay. Invasion assays using *Pf*FCR3 parasites revealed a range invasion inhibition activities for culture supernatant containing *Pf*RH5 and *Pf*CyRPA rIgG1 antibodies (**Figure 2**). Furthermore, invasion inhibition of antibodies during the initial screening of crude supernatants showed concentration-dependent effects for most antibodies (**Figure 2**). The potency of the antibodies was compared to the anti-AMA-1 polyclonal antibody BG98 by parallel line analysis (CombiStats 7.0). Relative potency estimates for each of the rIgG1 tested compared to anti-AMA-1 (BG98) ranged from 1 to 967 (median 20) for *Pf*RH5 and from 3 to 201 (median 27) for *Pf*CyRPA (**Supplementary Table 1**). All of the antibodies generated here were either as effective (relative potency of 1) or better at inhibiting invasion than BG98 (relative potency >1). Furthermore, the antibodies with the highest invasion blocking activity for *Pf*Rh5 and *Pf*CyRPA compared to BG98, were 967-fold and 201-fold more potent than BG98, respectively (see **Supplementary Table 1** for the full list of antibodies). It should be noted that the potency of these rhIgG1 clones is not directly comparable to BG98 as it is a polyclonal antibody mix and it is not possible to assess the potency of individual clones within the mixture to the mAbs generated here.

Interestingly, three anti-*Pf*RH5 clones (1D8#11, 14D5#250, 15D12#256) and one anti-*Pf*CyRPA clone (7B7#7) showed invasion inhibition activity in the invasion assays despite having no blocking activity by BLI (**Supplementary Figure 1**). Of those, 1D8#11 recognised a single overlapping peptide by peptide microarray (ISEEIDDKSEETDDETEEVEDSI). Invasion inhibition data along with BLI traces (**Supplementary Figure 1**) and microarray data (see **Supplementary Figure 2 and Supplementary Spreadsheet File**) were used to select clones for a more comparable assessment of mAb activity of the crude supernatants. Thirteen *Pf*RH5 mAbs and 10 *Pf*CyRPA mAbs displaying dilution effects in the initial screen were further evaluated, using the invasion assay, at defined antibody concentrations (16.4 µg/ml [112 nM], 3.28 µg/ml [22.5 nM], 0.656 µg/ml [4.5 nM]; data not shown) confirming concentration-dependent effects allowing further down selection.

**Figure 3:**
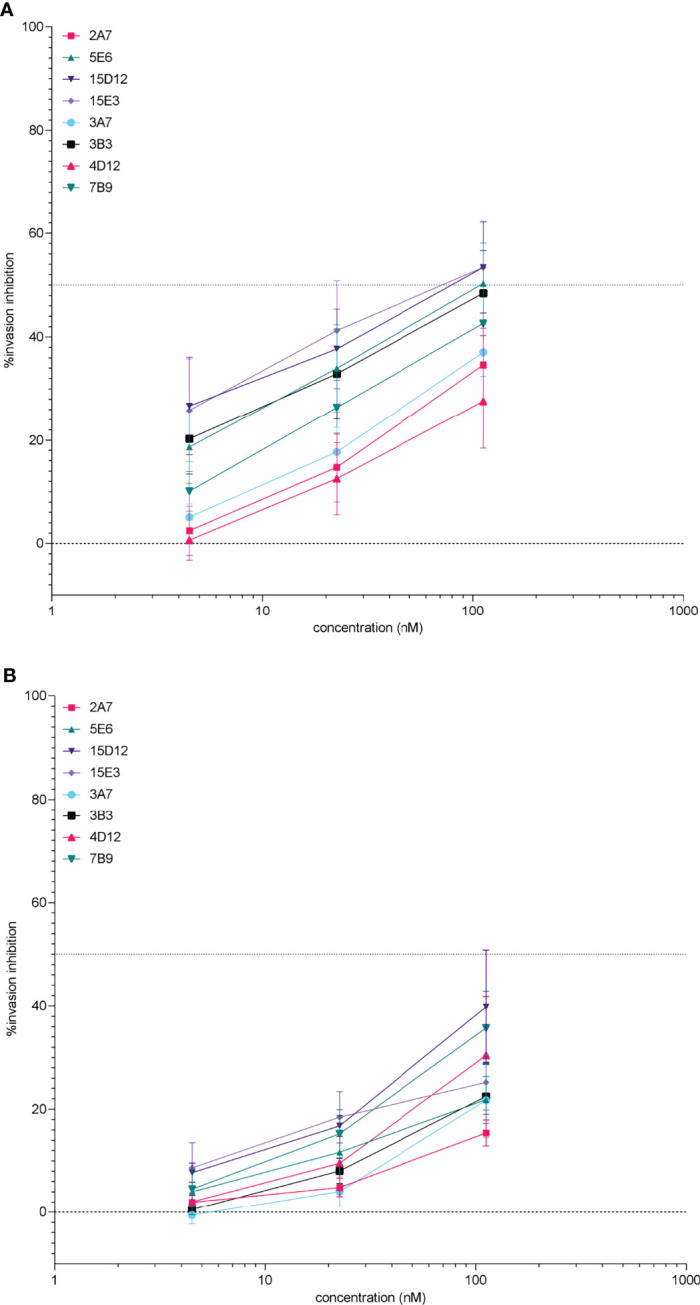
Potency of purified anti-*Pf*RH5 and -*Pf*CyRPA monoclonal antibodies on *P. falciparum* FCR3 **(A)** and NF54 parasites **(B)** after incubation during one intra-erythrocytic cycle. Invasion inhibition was determined by measuring parasitaemia by flow cytometry, percentage inhibition was calculated relative to the no antibody control wells. The percentage inhibition and standard error of the mean (error bars) are shown for 3 replicate experiments. Assays were performed using antibodies diluted to 112.3, 22.5, and 4.5 nM (i.e. 16.4, 3.28, and 0.656 µg/ml respectively) to facilitate comparisons with data using cell culture supernatants. Recombinant hIgG1 clones 2A7#70, 5E6#36, 15D12#256, and 15E3#259 are all directed against *Pf*RH5. Antibodies to *Pf*CyRPA are 3A7# 22, 3B3#17, 4D12#30, and 7B9#13.

Four anti-*Pf*RH5 clones and 4 anti-*Pf*CyRPA clones were selected for up-scaled production and purification. These purified mAbs retained invasion inhibition activity (**Figure 3**) and generally showed greater specificity by microarray with single or overlapping epitopes binding to the target antigen in the majority of cases (**Supplementary Figure 3**). Recombinant anti-*Pf*RH5 and anti-*Pf*CyRPA antibodies inhibited invasion in both *Pf*FCR3 and *Pf*NF54, but as can be seen in **Figure 3**, *Pf*FCR3 parasites were consistently more susceptible to rIgG1 invasion blocking activity. With the exception of clone 2A7#70 *Pf*RH5 rhIgG1 clones were significantly more inhibitory in *Pf*FCR3 compared to *Pf*NF54 (GLM_(1,3,2,2)_: *P <*0.001). All four purified *Pf*CyRPA rhIgG1 clones showed greater inhibition of invasion in *Pf*FCR3 (GLM_(1,3,2,2)_: *P <*0.001). Similarly, significant differences in replication rate between *Pf*FCR3 and *Pf*NF4 were observed for *Pf*RH5 rhIgG1 clones 5E6#36 (GLM_(2,1,2)_: *P* = 0.001) and 15E3#259 (GLM_(2,1,2)_: *P* = 0.001). No significant differences in replication rate were observed for rhIgG1 clones 2A7#70 (GLM_(2,1,2)_: *P* = 0.242) and 15D12#256 (GLM_(2,1,2)_: *P* = 0.068). For *Pf* CyRPA all rgIgG1 clones tested were significantly different between *Pf*FCR3 and *Pf*NF54; 3A7#22 (GLM_(2,1,2)_: *P* = 0.005), 3B3#17 (GLM_(2,1,2)_: *P <*0.001), 4D12#30 (GLM_(2,1,2)_: *P <*0.05), 7B8#13 (GLM_(2,1,2)_: *P* = 0.004), 5E6#36 (GLM_(2,1,2)_: *P* = 0.001), 15D12#256 (GLM_(2,1,2)_: *P* = 0.068), 15E3#259 (GLM_(2,1,2)_: *P* = 0.001) (**Supplementary Figure 4**).

### 3.3 Generation and Characterisation of PfCSP mAbs

The only available malaria vaccine, Mosquirix (RTS,S/AS01) includes the *Pf*CSP antigen, and new *Pf*CSP vaccines in development, so we next sought to produce anti-*Pf*CSP antibodies. For both chicken and rabbit immunisations a total of 128 panning reactions were performed resulting in 110 cloning reactions. Heavy and light chains were amplified and cloned into human IgG1 expression vectors, with a total of 50 panning reactions from chicken immunisations, and 76 from rabbit immunisations. Following transient expression, the presence of anti-*Pf*CSP antibodies in culture media was determined using ELISA. Eleven rIgG1 pools were positive; ~470 clones were selected of which 47 were bound to *Pf*CSP. From these, 19 clones were selected for sequencing and 7 unique sequence were identified; 3 were derived from chicken immunisations and 4 from rabbits.

**Figure 4:**
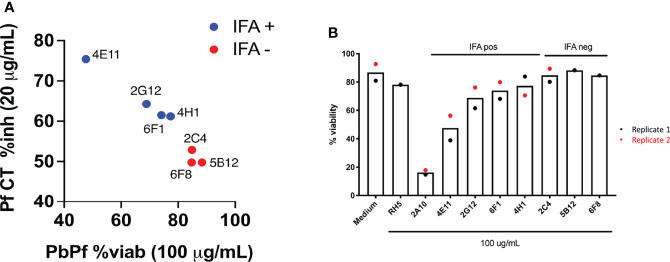
**(A)** Inhibition and cytotoxicity of recombinant IgG1 (rIgG1) against *Pf*CSP from cell culture supernatants. Cell traversal (CT) inhibition (y-axis) of *Pf*NF54 sporozoites is shown against viability of *P. berghei Pf*CSP transgenic parasites following incubation with the various mAbs. To note, cytotoxic activity in *PbPf*CSP sporozoites correlates with inhibition of *Pf* CT. Antibodies that do not bind to the *Pf*CSP on the sporozoite surface (red) are non-cytotoxic. IFA, immunofluorescence assay. **(B)** Viability of transgenic *P. berghei Pf* CSP parasites following incubation with cell culture supernatant containing recombinant anti-*Pf*CSP rIgG1. Controls included in the assay were cell culture medium alone and a recombinant anti-*Pf*RH5 rIgG1. The recombinant antibodies generated had a limited effect on sporozoite viability with 4E11#20 showing a moderate reduction in viability.

Using immunofluorescence and flow cytometry, 4/7 clones were found to bind to the surface of parasites (2G12#8, 4E11#20, 4H1#15 [rabbit] and 6F1#25 [chicken]; **Figure 4A**) and 3/7 displayed cytotoxicity and cell traversal inhibition profiles at 20 and 2 µg/ml (2G12#8, 4H11, 4E11#20, and 6F1#25; **Figure 4B** and **Table 2**).

**Table 2:** Summary of anti-*Pf*CSP mAb activity in sporozoite cell traversal (CT) assays.

Clone	CT assay (%blocking)		Binding of antibodies to sporozoite surface			Cytotoxicity (% viability)*
			Imaging	Flow Cytometry		
	Concentration		NF54 Pf SPZ	NF54 Pf SPZ	PbPf-GFP SPZ	PbPf-GFP SPZ (80 µg/ml)
	20 µg/ml	2 µg/ml				
2C4#2	52.9 (± 19.4)	40.4 (± 41.5)	**-**	**-**	**-**	98
2G12#8	64.3 (± 14.6)	50.5 (± 19.7)	**+**	**+**	**+**	79^#^
4H1#15	61.2 (± 9.9)	37.7 (± 7.2)	**+**	**+**	**+**	89
4E11#20	75.4 (± 12.1)	42.8 (± 21.2)	**+**	**+**	**+**	54
5B12#21	49.8 (± 9.5)	22.2 (± 15.5)	**-**	**-**	**-**	100
6F1#25	61.5 (± 11.3)	35.5 (± 2.4)	**+**	**+**	**+**	85
6F8#32	49.8 (± 16.1)	37.8 (± 15.0)	**-**	**-**	**-**	100
2A10			**+**	**+**	**+**	19
HybriMed	-9.8 (± 35.9)	**-**	**-**	**-**	**-**	100

Cell culture supernatants of seven recombinant human IgG1 clones were tested in cell traversal and cell toxicity assays. The mAbs were also used to confirm binding to native CSP expressed on the surface of P. falciparum NF54 sporozoites as well as on P. berghei chimeric for PfCSP. *Mean of two independent experiments for mAb recognizing non-permeabilised sporozoites. ^#^cytotoxicity measured at 60 μg/ml. + denotes positive surface staining of spozoites and – indicates no surface staining was observed.

To determine the range of epitopes present from the immunisations in rabbits and chickens the sera from the final bleeds was tested by peptide microarray. We found the major target was the *Pf*CSP NANP repeats (**Supplementary Figure 4**), with some additional reactivity against two additional regions outside of the NANP repeats. Sera from two of the immunised rabbits bound strongly to a 15-mer peptide (GQGHNMPNDPNRNVD). Sera from only one rabbit bound to an overlapping peptide (RNVDENANANSAVKNNNNEEPSDKHI). However, no clones were recovered from that animal. For the chicken immunisations, only sera from one chicken bound outside the NANP repeats to peptide EPSDKHIKEYLNKIQ but with no reactivity to the polymorphic sequences (EPSDKHI**T**EYLNKIQ and EPSDKHI**E**EYLNKIQ). Three clones were isolated from that immunization; 2C4#2, 6F1#25, 6F8#32).

## 4 Discussion

Here we describe the generation and initial characterisation of novel reference reagents to support malaria vaccine development. Through the EURIPRED project, we have produced, characterised and made available recombinant human IgG1 for the wider malaria research community. The recombinantly produced *Pf*RH5 and *Pf*CyRPA antibodies described provide a characterised and sequence defined material that can be used to standardise assays. Indeed, the antibodies produced inhibited invasion at similar concentrations (~16 µg/ml [~100 nm]) to some previously reported antibodies isolated from animals and humans (Douglas et al., 2011; Alanine et al., 2019; Knudsen et al., 2021). Furthermore, the activity of the some of the recombinant human antibodies described here appears to be comparable to monoclonal antibodies isolated from humans (Alanine et al., 2019) suggesting they have potential for development as biological standards.

We evaluated the antibodies to blood stage antigens in terms of relatively potency compared to an existing reference material (BG98). BG98 represents a large, but finite, supply of antibodies to *Pf*AMA-1 (Faber et al., 2013) and in this work also demonstrates the utility of a reference material. Indeed, the potency of the rhIgG1 clones described here at concentrations similar to previously reported *Pf*CyRPA and *Pf*RH5 antibodies (e.g. Douglas et al., 2011; Alanine et al., 2019; Knudsen et al., 2021) may make them more suitable as standards than BG98 which requires high concentrations to be used. In addition, independent testing to determine inter-laboratory variability of the antibodies was performed by GIA using *Pf*3D7. The relative antibody activity was consistent across laboratories (data not shown) highlighting that these materials can be used to harmonise laboratory tests globally. Indeed, the rIgG1 revealed similar potency of the antibodies as determined by rank order for inhibition (data not shown). Interestingly we observed increased sensitivity of *Pf*FCR3 to blocking antibodies relative to *Pf*NF54. The initial screening and selection steps were performed with only one laboratory isolate (*Pf*FCR3) which highlights, again, the value of standards as these differences can be identified and quantified. Strain differences in growth inhibition assays has previously been reported for both *Pf*RH5 (Douglas et al., 2011)and *Pf*CyRPA (Knudsen et al., 2021) despite low levels of polymorphisms in both antigens. Our data support these observations in additional laboratory isolates. Interestingly, we identified assay-dependent variability with the blocking antibodies, with *Pf*3D7 showing increased sensitivity to the rIgG1 in the invasion assay compared to GIA (data not shown); this has been found with other neutralising antibodies (Coxon et al., 2021) and is another reason why reference reagents are so valuable as they highlight that reliance on a single assay or strain can cause reproducibility issues and inconsistencies.

We observed few clones for the purified anti-*Pf*RH5 rIgG1 that recognised linear epitopes on the microarray. Conversely, most anti-*Pf*CyRPA rIgG1 bound to multiple epitopes on their target antigen but also on the other antigens present on the array. It is likely that conformational epitopes are critical for the binding and blocking activities of the rIgG1 described here. Indeed, it has previously been reported that many *Pf*RH5 epitopes are conformational (Alanine et al., 2019). This may explain why some of the antibodies for which we could not clearly identify linear epitopes were potent in inhibiting invasion (e.g. 3A7#22, 3B3#17, 4D12#30). Furthermore, the epitopes recognized by the purified rhIgG1 clones did not contain polymorphic sequences, suggesting that differences in antibody activity in *Pf*FCR3 and *Pf*NF54 are likely due to conformational epitopes. It will be interesting that given we found differences in invasion inhibition between strains, to fine tune epitope mapping to determine if these can be used to predict or confirm likely differences in antibody potency.

The materials evaluated were developed and purified as part of this work were prepared in relatively small quantity, but importantly the sequence and therefore the capability to prepare more are available. As these antibodies are recombinant they theoretically constitute an infinite resource ensuring a supply for the future. However, as a note of caution the route of production may be important as it is know that glycosylation patterns of immunoglobulins are important for functional activity (Schroeder and Cavacini, 2010; Subedi and Barb, 2015; van de Bovenkamp et al., 2016). Indeed, if we are proposing that the community uses these antibodies to harmonise and standardise assays, it is important that we understand the effects of post-translational modifications on the material and ensure we are using a defined and optimised production process. Indeed, work by our group and others (Ragotte et al., 2022) has already shown that the choice of cell expression system used in the production of the antibodies effects antibody activity and we currently are investigating this in more detail at NIBSC. Furthermore, it is known that post-translational modifications are important for the activity of biological molecules including glycosylation in the context of immunoglobulins (Schroeder and Cavacini, 2010; Subedi and Barb, 2015; van de Bovenkamp et al., 2016). Glycosylation can be influenced by many factors including the cells used for the production of antibodies and the additives used in cell culture media (Kim et al., 2018; Ehret et al., 2019). It is therefore important to consider production of recombinant monoclonal antibodies. Indeed, variations in the biological activity of antibodies may result not from their sequences but their glycosylation patterns.

All the antibodies described were tested individually. It is probable that further screening of the purified rIgG1 would enable the identification of synergistic effects with different antibody combinations as has been previously reported for *Pf*CyRPA (Knudsen et al., 2021) and *Pf*RH5 (Alanine et al., 2019) As *Pf*RH5 and *Pf*CyRPA form a complex during invasion, the presence of antibodies to both antigens could further inhibit invasion. Combining antibodies to the same target with different binding profiles could allow for the identification of more inhibitory antibody cocktails which would also enable the formulation of more robust standards. This approach would enable the formulation of polyclonal rIgG1 materials that may be more commutable and therefore comparable to the breadth of responses seen in individuals exposed to *P*. *falciparum*. In addition, many antibodies with high potency were not purified and further characterised, and these may warrant further investigation such as anti-*Pf*RH5 clone 18D8 #291 with >900-fold increased potency compared to anti-AMA-1 (BG98), or anti-*Pf*CyRPA clone 17H7 #73 with a potency 80-fold higher than anti-AMA-1 (BG98).

The antibodies described here also offer significant opportunities to generate new reference materials and research reagents as recombinant antibodies can be cloned into different immunoglobulin backbones. Specific subclasses of IgG are associated with *P. falciparum* infection (Dobano et al., 2019), namely IgG1 and IgG3. The system described here for the expression of human recombinant IgGs allows for the complementary determining regions (CDRs) to be cloned into other IgG subclasses (e.g., IgG3 framework) to determine how changing Fc functionality affects *in vitro* or *in vivo* activity at the two key stages of the parasite life-cycle (liver and blood). Altering the IgG subclass may enable the development of other reagents that more closely reflect the human immune response in assays and therefore, provide better indicators of candidate antigen performance during clinical development. These would also provide standardisation reagents more closely aligned to the biological targets of interest.

While the production of the *Pf*CSP antibodies was challenging we have successfully generated seven new monoclonal antibodies. The reasons for the difficulties in expression and isolation of the *Pf*CSP rIgG1 are not clear and further work is on-going with the clones generated. The microarray data showed that the polyclonal sera from one chicken and two rabbits bound to regions outside the NANP repeats. Three clones were isolated from the immunized chicken (2C4#2, 6F1#25, 6F8#32) and one clone from the rabbits (5B12#21). Determining what epitopes the purified rhIgG1 clones recognise by microarray will further allow characterisation of these mAbs. The antibodies generated did not show increased potency compared to 2A10 as crude cell supernatants. However, the data obtained from the crude supernatants is encouraging and all antibodies generated showed some activity in the various assays used (i.e. cell traversal inhibition, cell toxicity). For example, antibody 4E11#20 showed relatively high levels of activity in the cell traversal assay (75.45 ± 12.1% inhibition) and cytotoxicity (54% viability). Further work will be required on the purified rIgG1 to better understand their activity and potential as reference reagents and/or research reagents.

The materials described here are a first step in the provision of quality standards for malaria research that can support robust assay development and material characterisation for vaccines.

## 5 Reagents

The recombinant human monoclonal antibodies described herein are available from CFAR-NIBSC.

**Table:** 

NIBSC Catalogue #	Description
NR0025	Purified mAb anti-*Pf*CSP1 (4E11#20)
NR0026	Purified mAb anti-*Pf*CSP1 (5B12#21)
NR0027	Purified mAb anti-*Pf*CSP1 (6F1#25)
NR0028	Purified mAb anti-*Pf*CSP1 (2G12#8)
NR0029	Purified mAb anti-*Pf*CSP1 (4H1#15)
NR0030	Purified mAb anti-*Pf*CSP1 (2C4#2)
NR0031	Purified mAb anti-*Pf*CSP1 (6F8#32)
100851	Purified mAb anti-*Pf*RH5 (2A7#70)
100852	Purified mAb anti-*Pf*RH5 (5E6#36)
100853	Purified mAb anti-*Pf*RH5 (15D12#256)
100854	Purified mAb anti-*Pf*RH5 (15E3#259)
100855	Purified mAb anti-*Pf*CyRPA (3A7#22)
100856	Purified mAb anti-*Pf*CyRPA (3B3#17)
100857	Purified mAb anti-*Pf*CyRPA (4D12#30)
100858	Purified mAb anti-*Pf*CyRPA (7B9#13)

## Data Availability Statement

The VH/VL sequence were analysed and validated using the international ImmunoGeneTics (IMGT) information system https://www.imgt.org/.

## Ethics Statement

The animal study was reviewed and approved by Estonian National Board of Animal Experiments (No. 115, 07.09.2012; No. 87, 28.08.2007).

## Author Contributions

AN performed the invasion assays, blood-stage antibody selection, data analysis, project management, coordinated with partners for assays, and wrote the manuscript. GK performed antibody isolation by HybriFree technology and project management. RP performed the immunisations, Octet blocking assay, and project management. EJ did the antibody purification and antibody screening by ELISA. PH performed the microarray experiments. EA and RA performed the sporozoite cytotoxicity, cell traversal and immunofluorescence assays and corresponding figures. OS performed the sporozoite cell traversal assays. DQ performed GIA with the purified mAbs. YLD and MH provided critical logistics for the shipment and storage of the antibodies, and project management. UR led the work at JPT Technologies, designed the microarray slides, provided data insights and analysis. AT generated the monoclonal antibodies, and provided scientific insights. SJD provided reagents and input into the selection of the antigens. SG and MMH coordinated the EURIPRED grant. PWB conceived of the project, selected the antigens and provided project management. All authors reviewed the manuscript and agreed with the version submitted.

## Funding

This work was the European Union’s Seventh Framework Programme [FP7-INFRA-2012] under Grant Agreement No: 312661 - European Research Infrastructures for Poverty Related Diseases (EURIPRED). SD is a Jenner Investigator, a Lister Institute Research Prize Fellow, and held a Wellcome Trust Senior Fellowship (106917/Z/15/Z).

## Conflict of Interest

SJD is a named inventor on patent applications relating to *Pf*RH5 and/or other malaria vaccines, mAbs, and immunisation regimes. UR and PH are employed by JPT Peptide Technologies GmbH. GK, RP, EJ, and AT are employed by Icosagen Cell Factory.

The remaining authors declare that the research was conducted in the absence of any commercial or financial relationships that could be construed as a potential conflict of interest.

## Publisher’s Note

All claims expressed in this article are solely those of the authors and do not necessarily represent those of their affiliated organizations, or those of the publisher, the editors and the reviewers. Any product that may be evaluated in this article, or claim that may be made by its manufacturer, is not guaranteed or endorsed by the publisher.
