# Supplementary material for: Expanding the Malaria Antibody Toolkit: Development and Characterisation of Plasmodium falciparum RH5, CyRPA, and CSP Recombinant Human Monoclonal Antibodies
Source: Front Cell Infect Microbiol. 2022 Jun 16;12:901253. doi: 10.3389/fcimb.2022.901253 (PMC9243361; doi:10.3389/fcimb.2022.901253)

Supplementary Material

# Supplementary Figures and Tables

## Supplementary Table

**Supplementary Table 1**. Relative potency of the crude supernatants expressing recombinant human immunoglobulin 1 compared to polyconal anti-AMA-1 (BG98 standard).

| **Antigen** | **Clone** | **Potency v AMA-1 (fold increase)** |
| --- | --- | --- |
| RH5 | 10A12#118 | 13 |
| RH5 | 10D3#123 | 12 |
| RH5 | 10E7#133 | 12 |
| RH5 | 14B11#227 | 13 |
| RH5 | 14D5#250 | 18 |
| RH5 | 14F2#232 | 17 |
| RH5 | 14H8#252 | 8 |
| RH5 | 15A11#233 | 6 |
| RH5 | 15A4#238 | 24 |
| RH5 | 15B7#243 | 6 |
| RH5 | 15D12#256 | 499 |
| RH5 | 15E3#259 | 663 |
| RH5 | 15F4#240 | 8 |
| RH5 | 18D6 #266 | 197 |
| RH5 | 18D8 #291 | 967 |
| RH5 | 18F5 #274 | 82 |
| RH5 | 18H5 #270 | 54 |
| RH5 | 19F8 #282 | 473 |
| RH5 | 20C7#300 | 73 |
| RH5 | 23F4#307 | 46 |
| RH5 | 1A4#27 | 23 |
| RH5 | 1D8#11 | 1 |
| RH5 | 1E10#8 | 44 |
| RH5 | 1E2#22 | 9 |
| RH5 | 2A7#70 | 81 |
| RH5 | 2B4#32 | 25 |
| RH5 | 5C9#55 | 11 |
| RH5 | 5D6#170 | 14 |
| RH5 | 5E6#36 | 867 |
| RH5 | 5G12#46 | 80 |
| RH5 | 5H11#51 | 21 |
| RH5 | 6A8#87 | 5 |
| RH5 | 6D1#96 | 9 |
| CyRPA | 3A7#22 | 3 |
| CyRPA | 3B3#17 | 4 |
| CyRPA | 3E12#12 | 8 |
| CyRPA | 3G11#15 | 34 |
| CyRPA | 4D12#30 | 201 |
| CyRPA | 4G3#4 | 13 |
| CyRPA | 7B7#7 | 5 |
| CyRPA | 7B9#13 | 30 |
| CyRPA | 8C8#18 | 8 |
| CyRPA | 11D11#36 | 27 |
| CyRPA | 11F7#29 | 28 |
| CyRPA | 12D2#33 | 9 |
| CyRPA | 16C1#106 | 62 |
| CyRPA | 22C2#81 | 54 |
| CyRPA | 22D9#93 | 146 |
| CyRPA | 22D11#75 | 6 |
| CyRPA | 17H7 #73 | 80 |

## Supplementary Figures


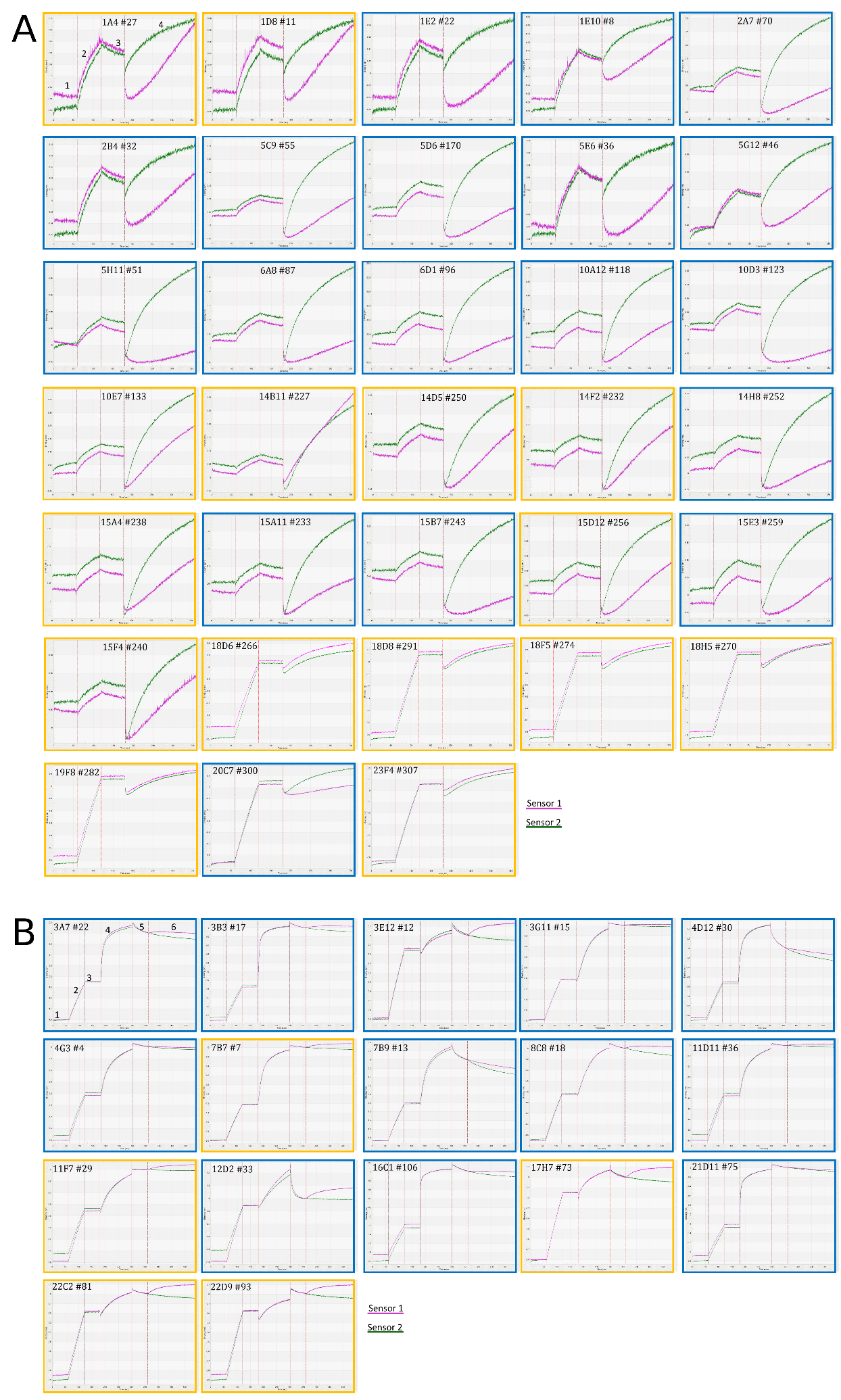


**Supplementary Figure 1.**

**Figure 1.** Biolayer interferometry traces of crude hIgG1 clones showing different blocking profiles for **(A)** *Pf*RH5 clones 1A4#27 and 5E6#36; and **(B)** *Pf*CyRPA clones 3A7#22 and 3G11#15). For *Pf*RH5 (A) traces show cell culture supernatant containing only *Pf*RH5 recombinant hIgG1 antibodies (green) and cell culture supernatant containing both recombinant *Pf*RH5 (*r*PfRH5) and recombinant human IgG1 antibodies (pink). Antibody 1A4#27 does not block *Pf*RH5 binding to *Pf*CyRPA. Conversely, antibody 5E6#39 blocks binding of *Pf*RH5 to *Pf*CyRPA (step 4). The numbered steps show 1) baseline for both sensors incubated in kinetic buffer; 2) incubation of the sensors with recombinant *Pf*CyRPA-biotin conjugate (*r*PfCyRPA); 3) baseline with the immobilised r*Pf*CyRPA-biotin in kinetic buffer; and 4) association of r*Pf*RH5 to *Pf*CyRPA. For *Pf*CyRPA (B) traces show incubation of one sensor in kinetic buffer (green) and incubation of the other sensor (pink) with *r*PfRH5 in association step (step 6). The numbered steps show 1) baseline readings for both sensors in kinetic buffer; 2) incubation with biotin-conjugated recombinant *Pf*CyRPA in kinetic buffer; 3) baseline readings for sensors with immobilised r*Pf*CyRPA in kinetic buffer; 4) saturation of the sensors with crude antibody cell culture supernatant containing specific anti-*Pf*CyRPA recombinant hIgG1s (pink); 5) baseline with immobilized *Pf*CyRPA-antibody complex in kinetic buffer; 6) One sensor incubated in kinetic buffer (green) and the second in kinetic buffer containing PfRH5 (purple). Clone 3G11#15 shows blocking activity (orange outline) while antibody clone 3A7#22 does not block binding of r*Pf*RH5 to r*Pf*CyRPA (blue outline). The y-axis shows binding (nm) and x-axis time (sec). Orange boxes indicate clones with no detectable blocking activity. Blue boxes denote clones with blocking activity as determined by biolayer interferometry.


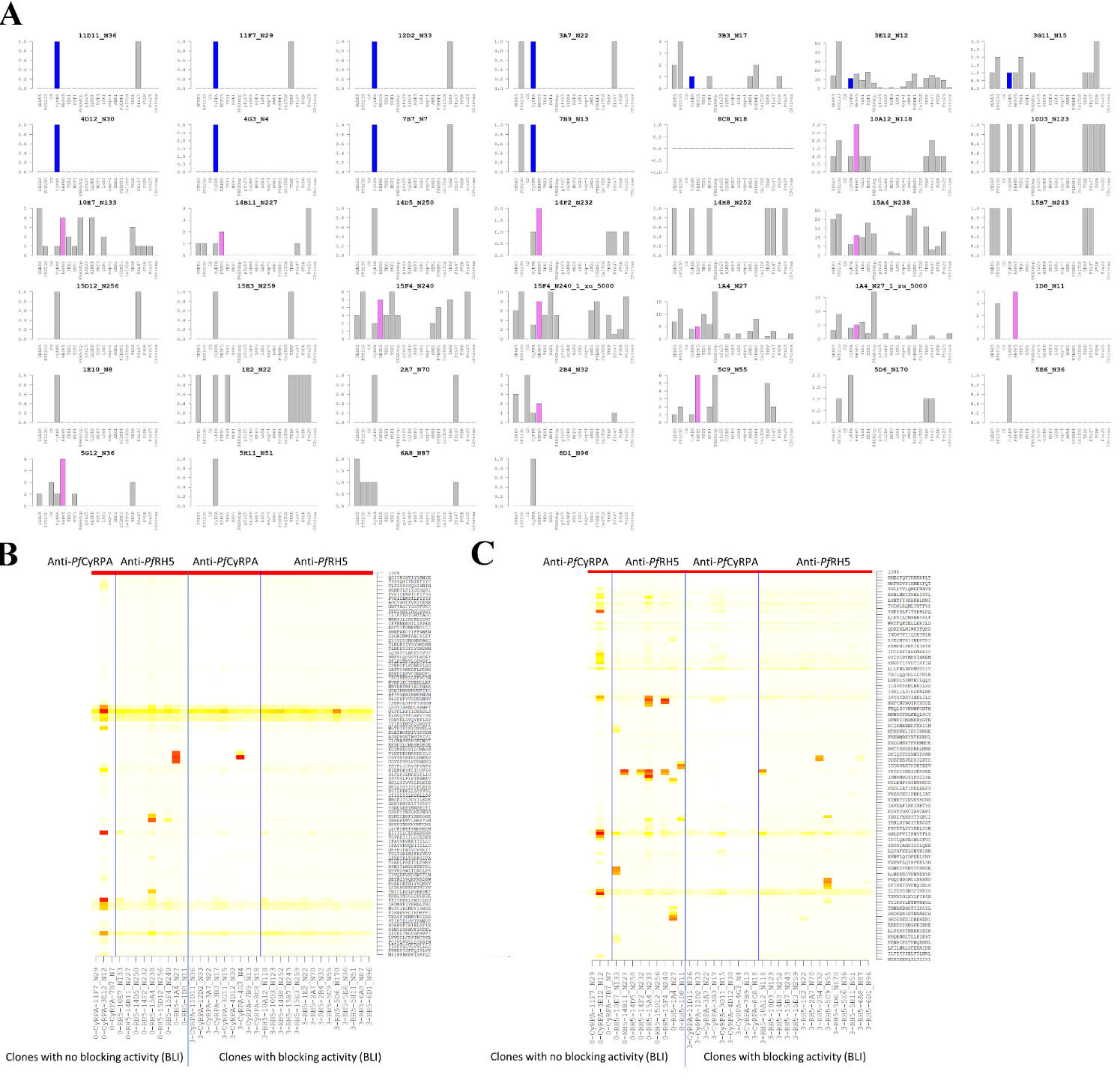


**Supplementary Figure 2.** Binding of crude cell supernatant expressing human recombinant IgG1 clones to linear peptide scans of malaria antigens on a peptide microarray. (A) Barplots of counted Signals (>10000) for each antigen. The target antigens for each sample are highlighted (blue: PfCyRPA; violet: PfRH5). (B) Antigen heatmap sorted by blocking activity as determined by biolayer interferometry for *Pf*RH5 showing binding of recombinant IgG1 monoclonals directed against PfRH5 and PfCyRPA. The clones are shown along the x-axis and overlapping peptides on the y-axis. Darker colours indicate brighter fluorescence signals in the array. C) Antigen heatmap sorted by blocking activity as determined by biolayer interferometry for *Pf*CyRPA. Recombinant IgG1 clones directed against *Pf*RH5 and *Pf*CyRPA are shown on the x-axis of the heatmap with the overlapping peptides shown on the y-axis. Darker colours indicate a brighter fluorescence signal in the array. Raw data are provided in a separate spreadsheet.

**
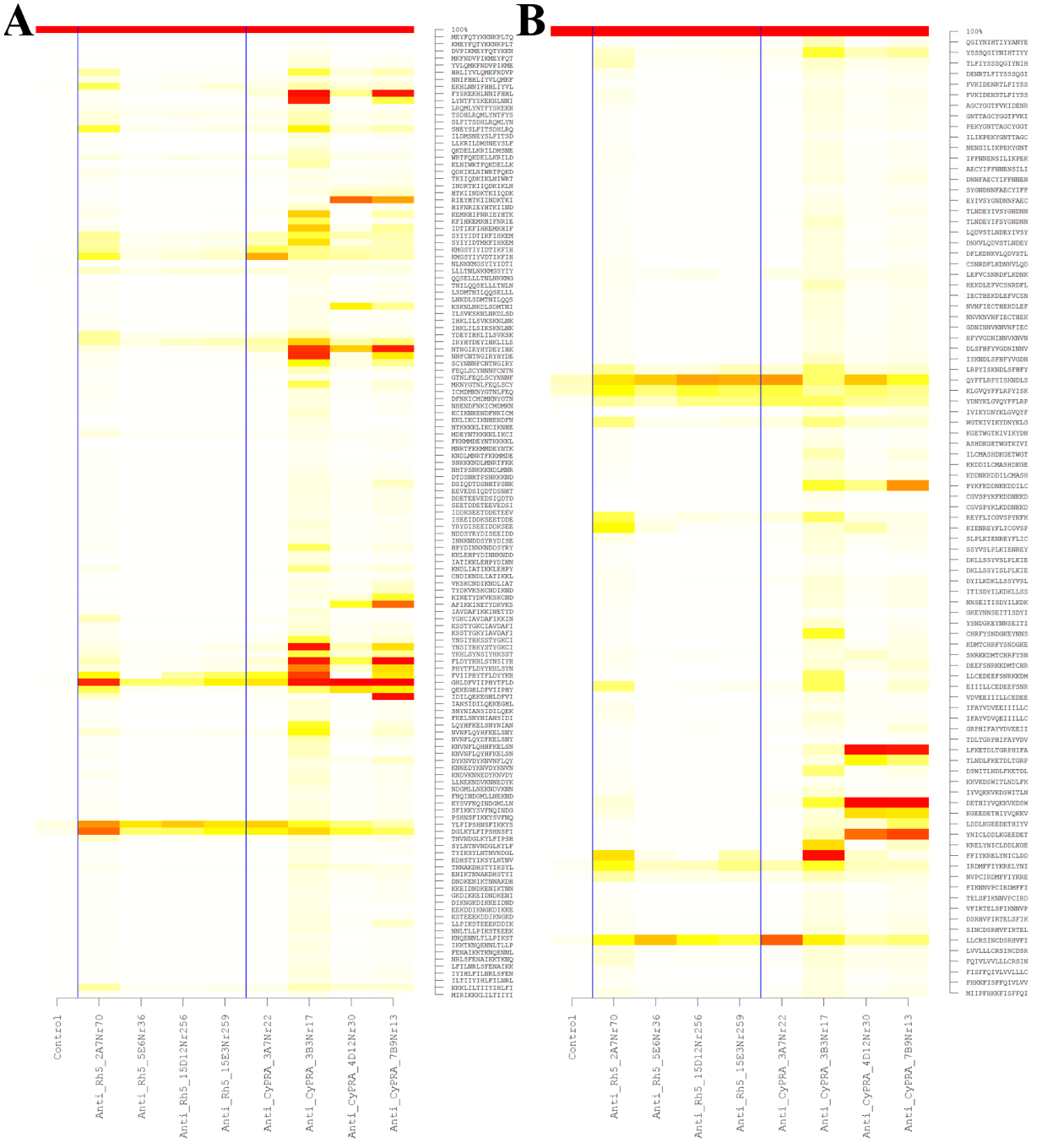
**

**Supplementary Figure 3.** Binding of human recombinant IgG1 clones to *Pf*CyRPA **(A)** and*Pf*RH5 **(B)**. Peptide array probed with down-selected purified recombinant IgG1. Darker colours on the heatmap indicate a brighter fluorescence signal in the array. **(A)** *Pf*RH5 antibody 2A7#70 bound strongly to linear epitopes of the *Pf*RH5 antigen. The other antibodies showed moderate binding to *Pf*RH5. PfCyRPA antibodies recognise several linear epitopes for *Pf*RH5. **(B)** Recombinant *Pf*CyRPA IgG1 clones bound strongly to recombinant *Pf*CyRPA. Clones 4D12#30 and 7B9#13 recognise similar epitopes.


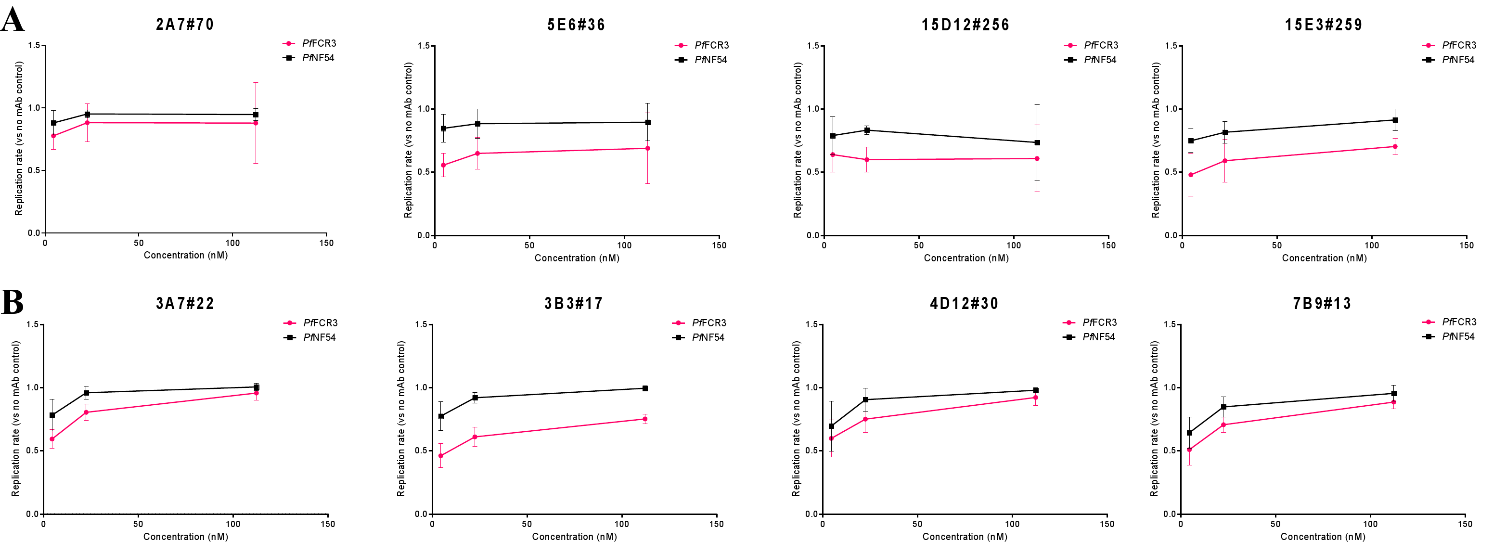


**Supplementary Figure 4.** Reduced replication rate of *P. falciparum* NF54 (*Pf*NF54) and *Pf*FCR3 parasites cultured with *Pf*RH5 and *Pf*CyRPA monoclonal antibodies for 1 cycle. Invasion assays were performed with antibodies diluted to 16.4, 3.28, and 0.656 µg/ml (112, 22, 5 nM) to facilitate comparisons with data using cell culture supernatants. Replication rate was calculated by dividing the end parasitaemia from the starting parasitaemia. Comparison of invasion inhibition of A) anti-*Pf*RH5 and B) anti-*Pf*CyRPA antibodies against *Pf*FCR3 (blue) and *Pf*NF54 (green). These data show the mean replication rate (and standard deviation) for each strain at different antibody concentrations (n= 3 experiments).

**Variable region sequence alignments**

Heavy and light chain variable region sequences and alignments for all clones generated are shown below, CDR-s are shown in blue.

***Pf*CyRPA**

Chicken host

Alignments of the variable light and heavy chain amino acid sequences of *Pf*CyRPA hrIgG1 clones 3A7#22, 3B3#17, 3E12#12, 3G11#15, 4D12#30, 4G3#4, 7B7#7, 7B9#13, 8C8#18, 11D11#36, 11F7#29, 12D2#33 were derived from chicken immunisations. Heavy chain and light variable region sequence alignments are shown below; CDR-s are highlighted in blue.


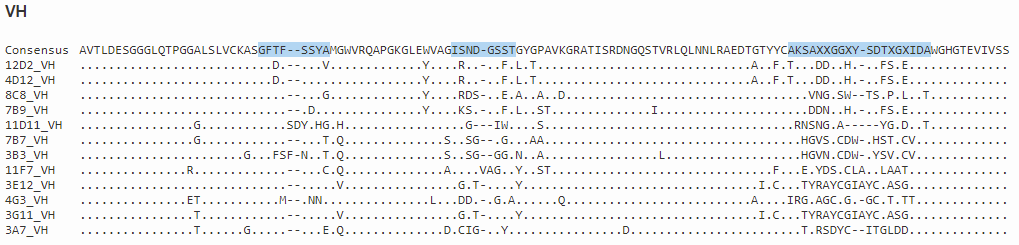


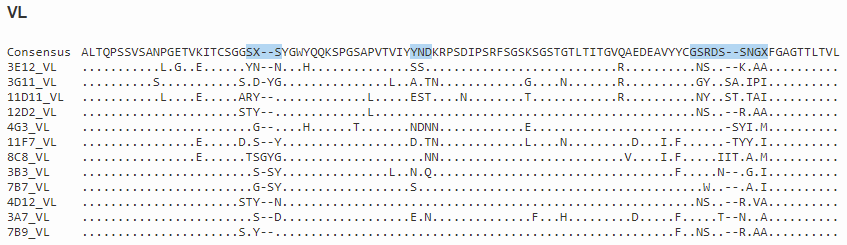


Rabbit host

*Pf*CyRPA clones 17H7#73, 16C1#106, 22C2#81, 21D11#75, and 22D9#93 were isolated from rabbit immunisations. Heavy chain and light variable region sequence alignments are shown below; CDR-s are highlighted in blue. Clone 22D9#93 did not contain a full variable heavy chain and is not included in the alignment. It is likely that this antibody binds the target antigen solely through the light chain.


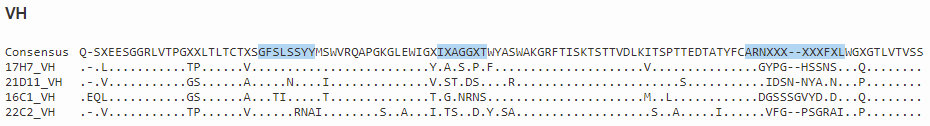


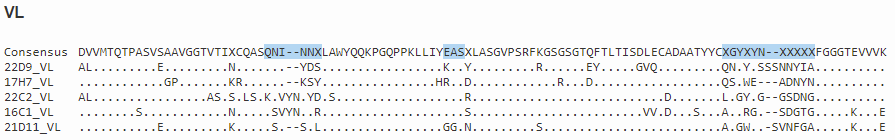


***Pf*RH5**

Chicken host

*Pf*RH5 rhIgG1 clones 1A4#27, 1D8#1, 1E10#8, 1E2#22, 2A7#70, 2B4#32, 5C9#55, 5D6#170, 5E6#36, 5G12#46, 5H11#51, 6A8#87, 6D1#96, 10A12#118, 10D3#123, 10E7#133, 14B11#227, 14D5#250, 14F2#232, 14H8#252, 15A11#233, 15A4#238, 15B7#243, 15D12#256, 15E3#259, and 15F4#240 were isolated from chicken immunisations. Heavy and light chain variable region sequences are shown below; CDR-s are highlighted in blue.


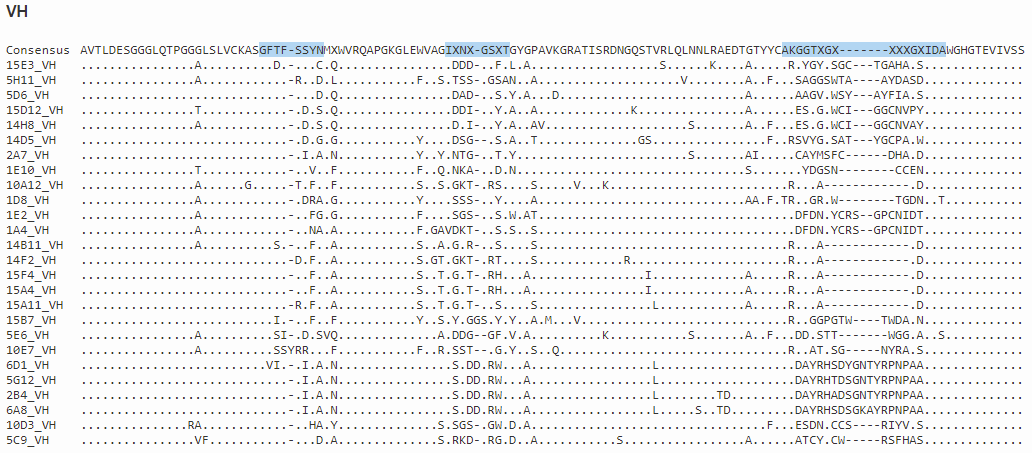


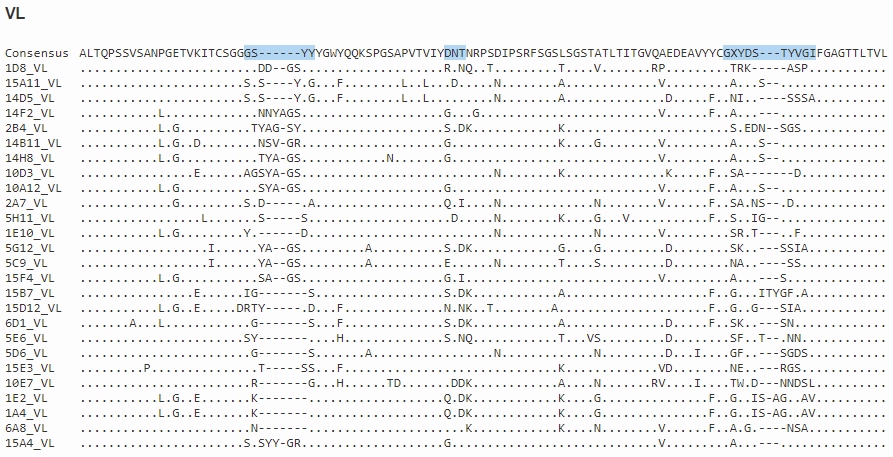


Rabbit host

*Pf*RH5 rhIgG1 clones 18D6 #266, 18D8 #291, 18F5 #274, 18H5 #270, 19F8 #282, 20C7#300, and 23F4#307 were isolated from rabbit immunisations. Heavy and light chain variable region sequence alignments are shown below; CDR-s are highlighted in blue.


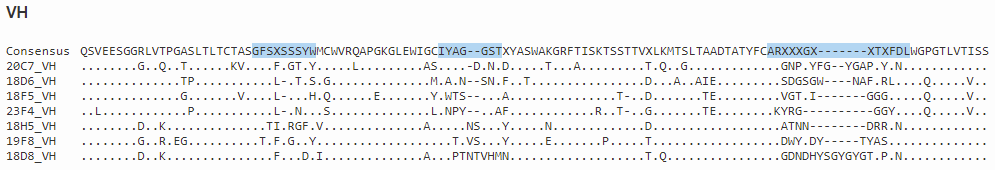


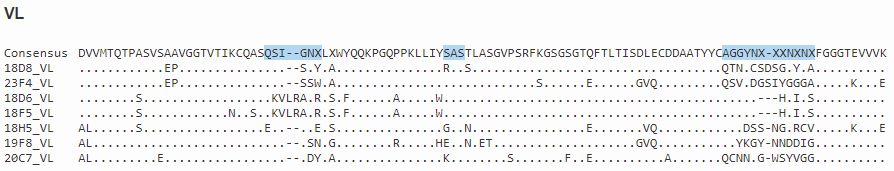


*Pf*CSP

Chicken host

Clones 2C4#2 6F1#25, and 6F8#32 were isolated from chicken immunisations. Heavy and light chain variable region sequences are shown below, CDR-s are highlighted in blue.


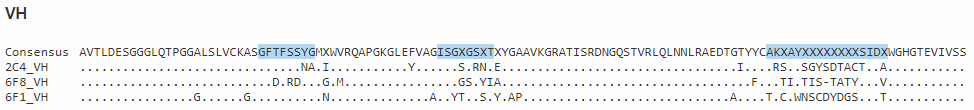


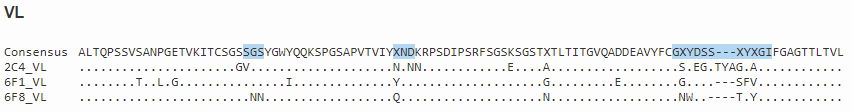


Rabbit host

Clones 2G12#8, 4E11#20, 4H1#15, and 5B12#21 were isolated from rabbit immunisations. Heavy and light chain variable region sequences are shown below, CDR-s are highlighted in blue.


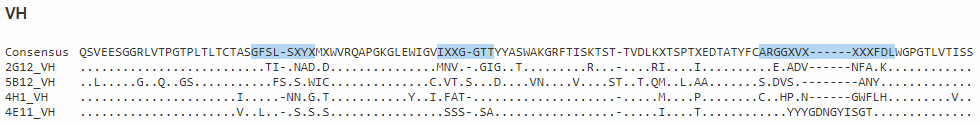


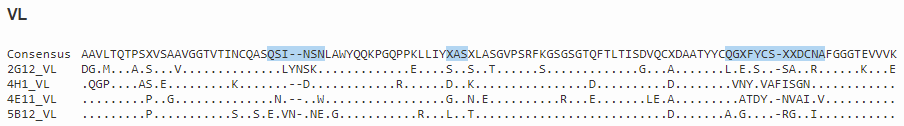

Supplement: Supplementary file 2 [file DataSheet_2.docx]
